# Decomposer diversity increases biomass production and shifts aboveground-belowground biomass allocation of common wheat

**DOI:** 10.1038/s41598-018-36294-3

**Published:** 2018-12-17

**Authors:** Nico Eisenhauer, Anja Vogel, Britta Jensen, Stefan Scheu

**Affiliations:** 1 German Centre for Integrative Biodiversity Research (iDiv) Halle-Jena-Leipzig, Deutscher Platz 5e, 04103 Leipzig, Germany; 2 Institute of Biology, Leipzig University, Deutscher Platz 5e, 04103 Leipzig, Germany; 3 Institute of Ecology and Evolution, University of Jena, Dornburger Straße 159, 07743 Jena, Germany; 4 J.F. Blumenbach Institute of Zoology and Anthropology, University of Göttingen, Untere Karspüle 2, 37073 Göttingen, Germany; 5 Centre of Biodiversity and Sustainable Land Use, University of Göttingen, Von-Siebold-Str. 8, 37075 Göttingen, Germany

## Abstract

Biodiversity is well known to enhance many ecosystem functions, but empirical evidence for the role of soil biodiversity for plant biomass production and allocation is scarce. Here we studied the effects of animal decomposer diversity (1, 2, and 4 species as well as a control without any decomposers) on the biomass production and aboveground-belowground biomass allocation of common wheat using two earthworm and two Collembola species using an additive design in two soil management types (organic and mineral fertilizer treatments) in a microcosm experiment. Shoot (+11%), spike (+7%), and root biomass (+56%), increased significantly with increasing decomposer diversity, and these effects were consistent across the two soil management types. Notably, decomposer diversity effects were stronger on root than on shoot biomass, significantly decreasing the shoot-to-root ratio (−27%). Increased plant biomass production was positively correlated with a decomposer richness-induced increase in soil water nitrate concentrations five weeks after the start of the experiment. However, elevated soil nitrate concentrations did not cause significantly higher plant tissue nitrogen concentrations and nitrogen amounts, suggesting that additional mechanisms might be at play. Consistent decomposer diversity effects across soil management types indicate that maintaining soil biodiversity is a robust and sustainable strategy to enhance crop yield.

## Introduction

Many studies have investigated the relationship between biodiversity and ecosystem functioning^1–3^ as well as the implications for the provisioning of ecosystem services^4,5^. There is broad consensus that biodiversity is important for the functioning of ecosystems^6^, while most empirical evidence is based on biodiversity effects of primary producers^7,8^. By contrast, there is limited knowledge of the effects of soil biodiversity on ecosystem functions^7,9,10^.

Soil biota represent an important component of terrestrial ecosystems by driving a variety of ecological functions and ecosystem services, such as nutrient cycling and decomposition processes^11,12^. Decomposer communities are the basis of “brown food webs” and depend on plant inputs entering the soil *via* roots, root exudates, and aboveground litter material^13,14^. Hence, plant growth and vegetation dynamics are indirectly affected by decomposers through changes in soil nutrient availability and distribution^15,16^. Therefore, ecosystem services, such as nutrient cycling, decomposition, and plant productivity, are influenced by interactions between organisms of above- and belowground sub-compartments^16–18^. Decomposers play distinct roles in the soil by facilitating different steps of decomposition processes, ranging from litter fragmentation to grazing on microbial communities^19^. As a consequence, interactions of functionally dissimilar soil organisms are likely to be critical for plant growth and crop yield^9,20^.

Plant growth is driven by the availability of resources and top-down pressure by herbivores and pests. Both of these bottom-up and top-down effects are affected by soil biodiversity^16,17^. Detritivores, such as earthworms and Collembola, drive nutrient mineralization processes by influencing the community composition and activity of soil microorganisms^21,22^ and plant antagonists by grazing and changes in soil conditions^23^. For instance, earthworms are known as ecosystem engineers by having numerous effects on soils like mixing soil layers, improving soil drainage and aeration, fragmenting and incorporating organic matter into the soil^21,24^, and influencing the composition and function of soil microbial and animal communities through various mechanisms^25,26^. Notably, different species and ecological groups of earthworms differentially affect ecosystem processes^27,28^. Epigeic earthworms reside mainly in the upper organic soil layers and feed on litter material, while endogeic earthworms live in the upper mineral soil, primarily consume organic material in mineral soil and form horizontal, non-permanent burrows. Anecic earthworms feed on litter but live in burrows in soil and incorporate litter from the soil surface into deeper soil layers. Collembola are abundant and diverse soil microarthropods, which play an important role in decomposition processes, regulate soil nutrient availability, and affect microbial activity and the structure of microbial communities^22,29,30^. Accordingly, earthworms and Collembola are important decomposers playing a key role in nutrient cycling and thus plant performance. Notably, previous studies have reported non-uniform effects of earthworms and Collembola on plant growth^31^, and that functional diversity of decomposers enhances nutrient mineralization, nutrient uptake by plants, plant complementarity effects, and community composition^32^, suggesting that the functional diversity of soil decomposer animals is critical for ecosystem functioning.

Although decomposers are known to enhance plant growth, their effects may be context-dependent. For instance, decomposer diversity effects have been shown to depend on plant community composition^31,33^. Moreover, decomposer diversity effects are likely to depend on soil conditions. A meta-analysis showed that earthworm effects on plant growth decrease with increasing levels of soil fertilization^34^. Effects of enhanced nutrient mineralization by decomposers may be superimposed by the addition of mineral fertilizers^34^, which is common practice in many agricultural fields, particularly if they are conventionally managed. However, such potential context-dependencies of decomposer diversity effects have rarely been tested thus far.

Here, we performed a microcosm experiment to test decomposer diversity effects of functionally dissimilar earthworm and Collembola species on the growth and above-belowground biomass allocation of common wheat (*Triticum aestivum*), one of the most relevant crop species worldwide. In order to explore potential mechanisms of decomposer diversity effects on plant growth, we repeatedly measured ammonium and nitrate concentrations in soil water, and determined nitrogen concentrations and amounts in plant tissue. Moreover, we tested decomposer diversity effects in two soils that differed in their management history with one having been treated with organic fertilizer and the other one with mineral fertilizer. We expected (1) decomposer diversity to enhance plant growth, but (2) decomposer diversity effects to be more pronounced in the organic soil than in the soil with mineral fertilizer.

## Results

**Figure 1:**
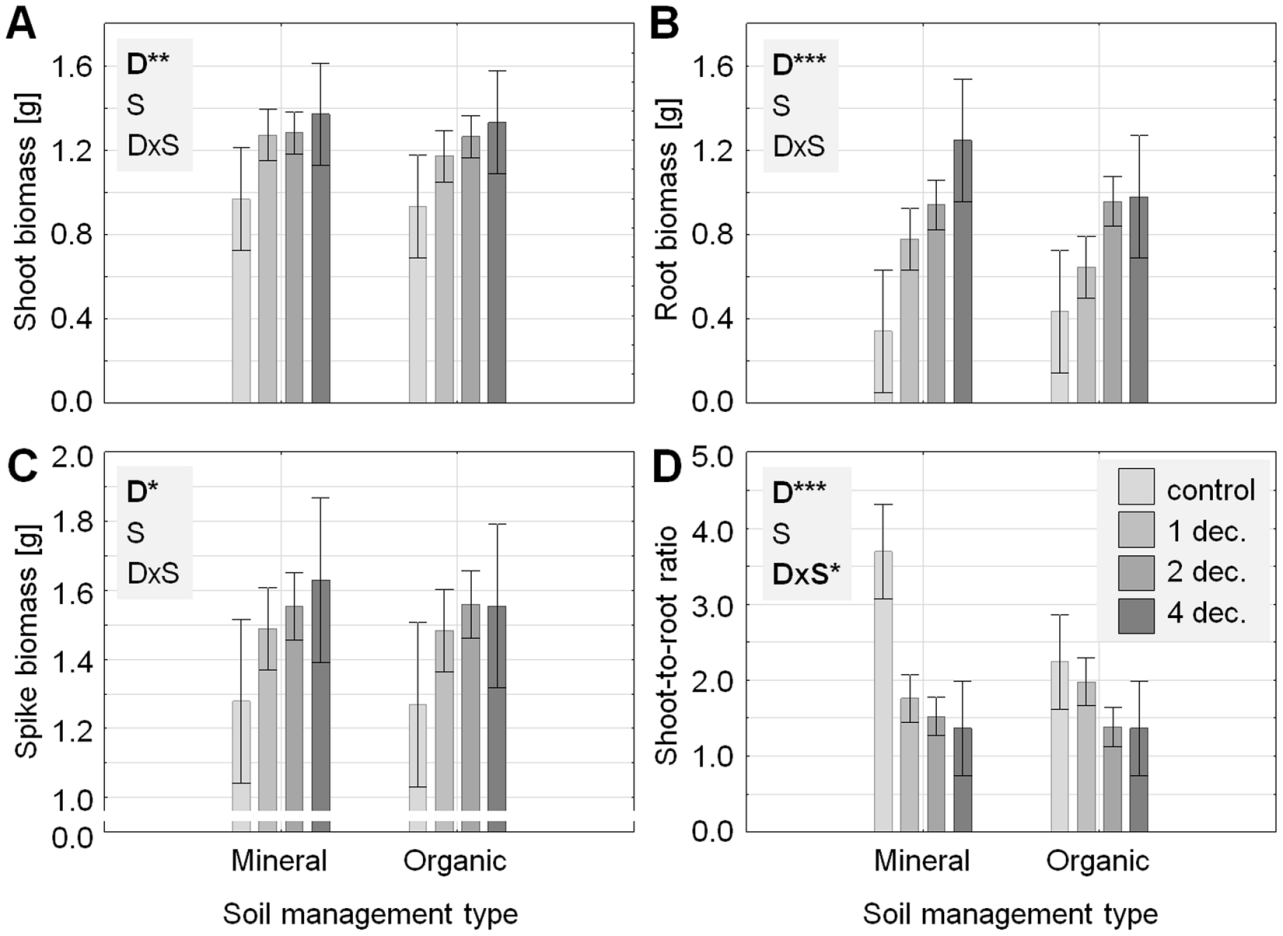
Effects of decomposer species richness (D: 0, 1, 2, and 4 species), soil management type (S: mineral *versus* organic fertilizer), as well as the interactions of both factors (D × S) on the biomass of shoots (**A**), roots (**B**), spikes (**C**) (all in g microcosm^−1^), and on shoot-to-root ratio (**D**) of common wheat. Significance levels: *P ≤ 0.05; **P < 0.01; ***P < 0.001. Means ± 95% confidence intervals.

Overall, our results demonstrate consistent decomposer effects across soil management types (Fig. 1; Table 1). Increasing decomposer species richness significantly enhanced the shoot, root, and spike biomass of common wheat, while effects of soil management type were non-significant (Fig. 1A–C; Table 1). Effects of decomposer species richness on plant growth were more pronounced on root than on shoot biomass (see absolute differences and 95% confidence intervals in Fig. 1B in comparison to Fig. 1A), causing a significant decrease of the shoot-to-root ratio with increasing decomposer species richness (Fig. 1D; Table 1). Moreover, decomposer species richness effects on the shoot-to-root ratio depended on soil management type, which was mostly due to significantly higher shoot-to-root ratio in the soil with mineral fertilizer in the absence of decomposers (see 95% confidence intervals in Fig. 1D).

**Table 1:** Table of F- and P-values of analyses of variance of the effects of block, decomposer diversity (0, 1, 2, and 4 species), soil management type (mineral *versus* organic), and the interaction between decomposer diversity and soil management type (D × S) on the shoot, root, and spike biomass of common wheat (all in g/microcosm), the shoot-to-root ratio, and nitrate concentration (%) in soil water five weeks after the start of the experiment.

	Block		Decomposer diversity		Soil management type		D × S	
	F-value	P-value	F-value	P-value	F-value	P-value	F-value	P-value
Shoot biomass	0.81	0.492	**4.63**	**0.005**	0.94	0.336	0.18	0.911
Root biomass	2.00	0.120	**12.98**	<**0.001**	0.72	0.399	0.95	0.422
Spike biomass	0.10	0.962	**3.62**	**0.016**	0.02	0.903	0.07	0.978
Shoot-to-root ratio	1.42	0.244	**14.81**	<**0.001**	0.87	0.352	**3.78**	**0.013**
Nitrate %, week 5	0.65	0.584	**6.97**	<**0.001**	**7.45**	**0.008**	1.47	0.227

Degrees of freedom: Block = 3, Decomposer diversity = 3, Soil management type = 1, D × S = 3. Significant effects are highlighted in bold.

**Figure 2:**
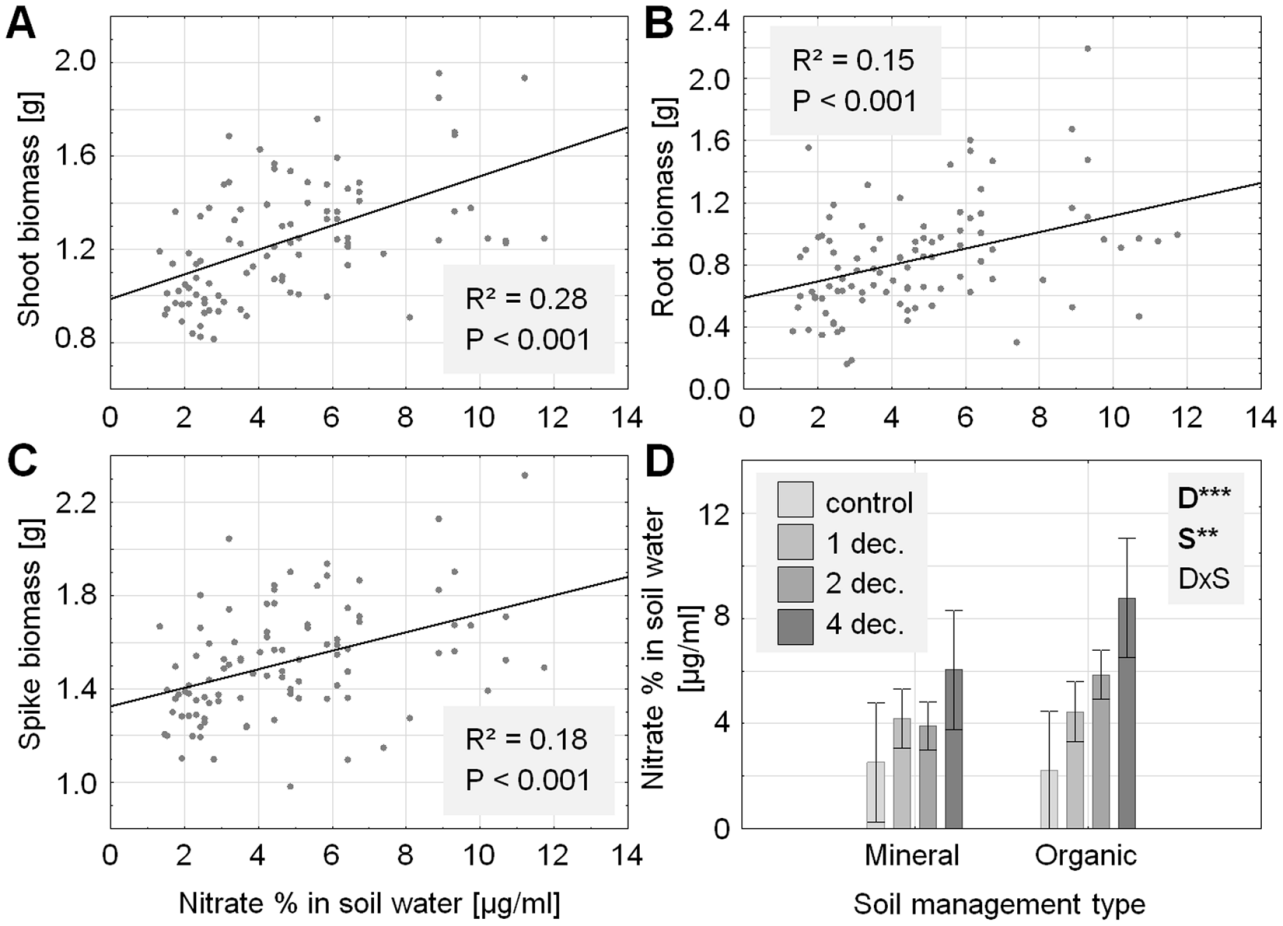
Correlations between nitrate concentrations in soil water five weeks after the start of the experiment and shoot (**A**), root (**B**), and spike biomass (**C**) of common wheat (all in g microcosm-1). (**D**) Effects of decomposer species richness (0, 1, 2, and 4 species) and soil management type (mineral *versus* organic fertilizer) on nitrate concentrations in soil water five weeks after establishment of the experiment. Significance levels: **P < 0.01; ***P < 0.001. Means ± 95% confidence intervals.

In order to explore potential mechanisms of decomposer species richness effects on plant growth, relationships between ammonium as well as nitrate concentrations in soil solution and plant biomass were investigated. These analyses showed that plant biomass was significantly positively correlated with nitrate concentrations in soil water five weeks after the start of the experiment (Fig. 2A–C; Table 2). Indeed, nitrate concentrations were significantly affected by the experimental treatments, being increased with increasing decomposer species richness and in the soil with organic fertilizer (Fig. 1D; Table 1). However, nitrogen concentrations, carbon-to-nitrogen ratio, and total amount of nitrogen in plant tissue were not significantly affected by the experimental treatments (all P > 0.09).

**Table 2:** Correlations plant biomass and ammonium and nitrate concentrations (%) in soil solution 2, 5, 8, and 11 weeks after the start of the experiment.

	Shoot biomass		Root biomass		Spike biomass		Shoot-to-root ratio	
	R²	P-value	R²	P-value	R²	P-value	R²	P-value
Ammonium %, week 2	<0.01	0.631	<0.01	0.896	<0.01	0.939	<0.01	0.670
Ammonium %, week 5	<0.01	0.481	<0.01	0.640	0.02	0.125	0.03	0.123
Ammonium %, week 8	<0.01	0.396	0.03	0.113	<0.01	0.882	<0.01	0.731
Ammonium %, week 11	<0.01	0.879	<0.01	0.593	0.02	0.160	<0.01	0.813
Nitrate %, week 2	<0.01	0.428	<0.01	0.826	<0.01	0.503	<0.01	0.535
Nitrate %, week 5	**0.28**	<**0.001**	**0.15**	<**0.001**	**0.18**	<**0.001**	0.02	0.167
Nitrate %, week 8	0.03	0.122	<0.01	0.448	0.02	0.140	<0.01	0.709
Nitrate %, week 11	0.01	0.318	0.02	0.191	0.01	0.291	0.02	0.141

Significant correlations are highlighted in bold.

## Discussion

The present study underlines the importance of soil biodiversity for the functioning of ecosystems and crop yield. While our hypothesis (1) was confirmed, stating that decomposer diversity increases plant yield, our hypothesis (2) was not confirmed, stating that decomposer diversity effects on plant yield differ between soils with different management history. Rather, we found consistent decomposer diversity effects on plant growth across soil management types, which most likely were mediated in part by elevated nutrient mineralization rates at high decomposer diversity (although the total amount of nitrogen in plant tissue was not significantly affected by the experimental treatments). Moreover, our results show that common wheat changed above-belowground biomass allocation in response to decomposer diversity with relatively higher root biomass at high decomposer diversity. These results may suggest that decomposer diversity not only enhances crop yield, but potentially also the resistance of common wheat against climate extremes like drought, as high root biomass and changes in root traits can mitigate detrimental climate effects^35^.

The diversity of soil organisms is likely to drive important ecosystem functions and services^11,12^. The soil food web is primarily fueled by the “brown energy channel”, i.e., decomposers like microorganisms and detritivore animals that process dead organic material. Further, decomposers affect important ecosystem functions including plant growth. Here we used functionally dissimilar earthworm (an anecic species that mostly feeds on aboveground litter material and forms vertical burrows in soil and an endogeic species that feeds on organic material in the soil and forms horizontal burrows) and Collembola species (a hemiedaphic species that feeds on litter, organic material, and microorganisms in the top soil and at the soil surface, and an euedaphic species that mostly feeds on organic material and microorganisms in the soil) to test decomposer diversity effects on the biomass allocation of common wheat. Our results show that decomposer diversity enhanced nitrate availability in soil water during a critical phase of plant growth (five weeks after the start of the experiment), which correlated significantly with plant biomass at the end of the experiment. However, this did not translate into elevated nitrogen concentrations and total nitrogen amounts in plant tissue at the end of the experiment. Nevertheless, these findings suggest that decomposer diversity can increase crop yield by enhancing nutrient mineralization and availability for plants.

The experimental design did not allow to test for decomposer identity effects, which likely co-determined significant diversity effects^9^. However, multiple 2- and 4-species mixtures had higher plant biomass and nitrate concentrations in soil water than the most productive monoculture treatment, suggesting synergistic effects of decomposer diversity^20^ and transgressive overyielding^36^. We do not present the respective results though and call for future research on additive *versus* non-additive decomposer diversity effects, as we were unable to keep the total decomposer biomass of all treatments equal given the much higher individual body mass of earthworms compared with Collembola, because this would have resulted in unnaturally high Collembola abundances^9^. Thus, the present experimental design does not allow to fully separate decomposer diversity from decomposer idenity and abundance effects, which all are important aspects of decomposer community composition.

In line with our expectations, we found some support that decomposer diversity effects were mediated *via* elevated nutrient availability in the soil (nitrate concentrations in soil water), although nitrogen concentrations and amounts in plant tissue at the end of the experiment did not support our assumption. Moreover, in contrast to our hypothesis, the decomposer diversity effects were not altered by the soil management types. We expected decomposer effects to be less pronounced in soil that had been treated with mineral fertilizer for many years^34^. In fact, nitrate concentrations in soil water were even higher in the organically treated soil rather than in the conventionally managed soil 5 weeks after the start of the experiment, suggesting that organic fertilizer may have longer-lasting effects than mineral fertilizer on plant growth. Accordingly, decomposer diversity effects tended to be more pronounced in the organically treated soil, but the difference to the conventionally managed soil was not significant. In general, addition of organic fertilizers/subsidies has been shown to increase the abundance of decomposer animals in comparison to agricultural fields treated with mineral fertilizers^37,38^, which may enhance decomposition and nutrient mineralization rates in a sustainable way.

Decomposer diversity did not only enhance wheat yield, but also decreased the shoot-to-root ratio, because decomposer diversity effects were more pronounced on root than on shoot biomass. This finding might have important implications for the stability of crop yield. Higher root biomass is likely to increase plant nutrient capture as well as water uptake from the soil and might be one important prerequisite for higher resistance of plants during detrimental environmental conditions, such as heavy storms and drought events^39,40^. Previous studies have shown that Collembola community composition can alter the vertical distribution of plant roots depending on plant functional group identity^33^, and decomposer diversity can increase deep root biomass^32^, e.g., by creating burrows allowing deeper root penetration. Unfortunately, we did not explore the depth distribution of plant roots in the present study, but future experiments should investigate potential decomposer diversity effects on roots in different soil depths as well as measure plant physiological responses. Moreover, given that we were only able to investigate the effects of a relatively short decomposer diversity gradient in the present study, future research should explore if decomposer diversity effects saturate the closer the community comes to the carrying capacity (both in terms of abundance and species richness) of the respective system. Thus, combined approaches of controlled community assembly experiments^41^, such as done in the present experiment, together with the study of the effects of the removal of taxa from previously intact communities and natural soil biodiversity gradients^42,43^ may provide additional insights into the significance of decomposer diversity. Notably, such studies should not only focus on one focal function, but on the multifunctionality of the system^8^.

Taken together, our present results suggest that decomposer diversity may enhance crop yield and its stability across different environmental settings (although organic agriculture and resulting higher biodiversity may not always increase crop yield stability^44^), adding to the notion that soil biodiversity is important for many ecosystem functions^41–43^. This suggests that a sustainable land use supporting high soil biodiversity^12^ may compensate for some of the yield loss of less intensive management.

## Methods

### Experimental setup

We varied the number of decomposer species in differently managed soil types and analyzed the biomass of common wheat in a microcosm experiment. The soil was derived from a weakly haplic luvisol (sL) (typic Hapludalf) on deep deposits of alluvial loess in the canton Basel (Therwil, Switzerland). The soil was taken from the ‘DOK’ project, a field experiment comparing organic and conventional agricultural farming systems for the growth of different crop species^45,46^. It was set up by the Agroscope Reckenholz-Tänikon Research Station (ART) and the Research Institute of Organic Agriculture (FiBL) in 1978 at Therwil (7°33′E, 47°30′N). The climate is relatively dry and mild with a mean annual precipitation of 785 mm and a mean annual temperature of 9.5 °C^47^. In May 2005, we collected soil from multiple plots of two contrasting soil farming systems that had been managed in the frame of the DOK long-term trial. The soils per management type were mixed to have one bulk sample for each management type. One soil management type was bio-organic receiving rotted farmyard manure and aerated liquid manure (1.4 fertilizer produced per livestock unit) as organic fertilizer with a substitute of rock powder and Kalimagnesia (FiBL Dossier 2000) for biological pest control. The second soil management type used mineral NPK fertilizer and chemical pest control^37,45^. Both soils had a pH of 6, with soil organic matter in the bio-organic treatment being slightly higher than in the conventional farming system^48^. The soil contained residues of silage maize, which was the last crop before sampling. The seven-year crop rotation was the same for both farming systems and included potatoes, wheat, soybean, grass/clover, and maize. More details on the experimental design, the different fertilizer treatments, and crops can be found in Mäder *et al*.^37^.

After sampling, the soil was defaunated by freezing (−20 °C, nine months), and homogenized by sieving (1 cm mesh size). Each microcosm was filled with 1.3 kg of soil (fresh weight). The microcosms consisted of PVC tubes (inner diameter 10 cm, height 20 cm), which were closed at the bottom. PVC foliage was taped around the upper edge (10.5 cm height) to prevent the animals from escaping or colonizing^33^. This size of microcosms was chosen, because it was proven useful to study plant-decomposer interactions and underlying mechanisms under laboratory conditions^20,31–33^, manipulating decomposer diversity under field conditions in such a controlled way is impossible, and because this approach allowed us to study soil water nitrogen concentrations using a suction cup system. Soil water was constantly collected in separate glass bottles *via* ceramic suction cups at the bottom of each microcosm using a vacuum pump (−400 to −500 hPA), allowing to collect leaching water for analyzing nutrient concentrations. Briefly, each microcosm contained a suction cup that was connected to a separate glass bottle *via* a hose. Each glass bottle was also connected to a common hose system that was connected to a vacuum pump that exerted an under-pressure to the whole hose system.

Two wheat seeds (*Triticum aestivum* L.; common wheat) were sown in each microcosm and watered as needed. One week later, the height of all plants was measured, and individuals with similar size were kept in the microcosm, whereas other individuals were removed to end up with one wheat individual per microcosm. Weeds and aphids occurring during the experiment were removed by hand. In addition, every plant individual received two larvae of *Chrysoperla carnea* (Katz Biotech AG, Baruth) to reduce aphid infestation.

Decomposers were represented by two earthworm and two Collembola species. The anecic earthworm species *Lumbricus terrestris* L. and the endogeic *Aporrectodea caliginosa* (Savigny) were extracted from meadows of the Jena Experiment^49^ and were kept in nutrient-rich loamy soil containing loess for several months at 6 °C. To acclimate earthworms to the experimental soil, they were kept in soil of the DOK project for two weeks before the start of the experiment^50^. Earthworm treatments received one individual of *A. caliginosa* (0.65 ± 0.22 g fresh weight without gut content) and/or *L. terrestris* (0.40 ± 0.22 g), i.e. microcosms with two earthworm species contained two earthworm individuals. By representing two different ecological groups of earthworms, these two species were supposed to have dissimilar effects. As anecic species, *L. terrestris* mostly feeds on aboveground litter material and forms vertical burrows in soil, while the endogeic species *A. caliginosa* feeds on organic material in soil and forms horizontal burrows.

Both Collembola species, hemiedaphic *Heteromurus nitidus* (Templeton 1835) and euedaphic *Supraphorura furcifera* (Stach 1901) were reared in laboratory cultures with baker´s yeast and distilled water at 18 °C. One week before the start of the experiment, microcosms with one Collembola species received ten individuals and microcosms with two species received 20 individuals. Moreover, in week five and seven another 10 individuals per species were added to ensure successful establishment of Collembola populations. Similar to the earthworm species, also the two Collembola species have different life history traits. *Heteromurus nitidus* (family Entomobryidae) is a pigmented, hemiedaphic species with a size of up to 3 mm and a well-developed furca^51^. *Supraphorura furcifera* (family Onychuiridae) is an unpigmented, euedaphic species with a size up to 1.4 mm and a reduced furca.

The decomposer species were combined in three diversity levels (1, 2, or 4 species) in eleven communities: each species alone (four single-species treatments), all possible pairs (six two-species communities), and all four species together. As control treatment, we set up microcosms without decomposers. All treatments were established in soil originating from organically and conventionally fertilized fields (two fertilizer/soil management treatments). We set up four replicates ending up with a total of 96 microcosms. The microcosms were arranged in four blocks, each block with one replicate per decomposer community x soil type combination, and blocks were randomized twice during the experiment (week four and eight).

The experiment was performed in a glasshouse under controlled conditions (20/18 °C day/night, 70% relative air humidity). In addition to daylight, we set up lamps (Powertonic Agro, Hochdruck Natriumdampflampe, 400 Watt, Philips) from above to enhance plant growth (as done before^33^) in a 16/8 hour day/night rhythm. Light intensity varied between 617 (±122) µMol m^−2^ s^−1^ on sunny and 462 (±142) µMol m^−2^ s^−1^ on cloudy days. The microcosms were irrigated with 50 ml deionized water daily or 100 ml every second day.

### Harvest and measurements

After 2, 5, 8, and 11 weeks, soil water collected in glass bottles *via* ceramic suction cups was analyzed for nutrient concentrations. Per microcosm, 100 ml soil water was collected and stored at 6 °C in the fridge until further processing. Ion-selected electrodes (Win Lab, Windaus, Germany) were used to measure ammonium and nitrate concentrations.

After 13 weeks, the shoot material of the plants was cut at the soil surface, and spikes were separated from stems and leaves. Roots were washed from the soil using a 2 mm sieve. Shoot, spike, and root material were dried separately at 60 °C to constant weight in a drying oven. Based on dry weight of shoot and root material, we calculated shoot-to-root ratio. Approximately 3 mg of powdered plant shoot and root material were weighed into tin capsules. Total carbon and nitrogen concentrations were determined by a coupled system consisting of an elemental analyzer (NA 1500, Carlo Erba, Milan) and a gas isotope mass spectrometer (MAT 251, Finngan^52^).

To check if the decomposer treatments were successfully established, earthworms were collected during root washing, and Collembola were extracted from soil cores (10 cm depth, 5 cm diameter) by heat^53^. All microcosms contained the target decomposer community at the end of the experiment and were thus included in the statistical analyses.

### Statistical analyses

The effects of block (1, 2, 3, 4), decomposer species richness (0, 1, 2, 4 species), soil management type (mineral *versus* organic), and the interaction between soil management type and decomposer species richness on shoot biomass, root biomass, spike biomass, and shoot-to-root ratio of common wheat were analyzed using sequential analyses of variance (type I sum of squares). Sequential analyses were performed to account for potential block effects, and block was always fitted first in the models. In addition, we performed correlations between shoot biomass, root biomass, spike biomass, as well as shoot-to-root ratio and ammonium and nitrate concentrations in soil water 2, 5, 8, and 11 weeks after the start of the experiment. As correlations between plant biomass and soil nitrate concentrations were only significant 5 weeks after the start of the experiment (Table 2), we also tested the effects of block, decomposer species richness, soil management type, and the interaction between decomposer species richness and soil management type on nitrate concentrations in soil water after 5 weeks as well as on nitrogen concentrations, carbon-to-nitrogen ratio, and total amount of N in plant tissue. All statistical analyses were performed with the statistical software Statistica 13.
